# Acknowledgement to Reviewers of *IJERPH* in 2016

**DOI:** 10.3390/ijerph14010065

**Published:** 2017-01-11

**Authors:** IJERPH Editorial Office

**Affiliations:** MDPI AG, St. Alban-Anlage 66, 4052 Basel, Switzerland; ijerph@mdpi.com

The editors of *IJERPH* would like to express their sincere gratitude to the following reviewers for assessing manuscripts in 2016.

We greatly appreciate the contribution of expert reviewers, which is crucial to the journal’s editorial process. We aim to recognize reviewer contributions through several mechanisms, of which the annual publication of reviewer names is one. Reviewers receive a voucher entitling them to a discount on their next MDPI publication and can download a certificate of recognition directly from our submission system. Additionally, reviewers can sign up to the service Publons (https://publons.com) to receive recognition. Of course, in these initiatives we are careful not to compromise reviewer confidentiality. Many reviewers see their work as a voluntary and often unseen part of their role as researchers. We are grateful to the time reviewers donate to our journals and the contribution they make.

If you are interested in becoming a reviewer for *IJERPH*, see the link at the bottom of the webpage http://www.mdpi.com/reviewers.

The following reviewed for *IJERPH* in 2016:
Aarabi, BizhanHawkins, MarquisPeng, Yen-PingAaseth, JanHayden, JoannaPengo, MartinoAasvang, GunnHayes, AlisonPenm, JonathanAbballe, AnnalisaHayes, Michael EdwardPennbrant, SandraAbbasian, FirouzHayman, Laura L.Pennock, NathanAbdin, EdimansyahHe, CongrongPepper, Jessica K.Abdul Aziz, AmmarHe, Li-MinPereira, AlcidesAbiGhannam, NiveenHe, QinghuaPérez, GlòriaAbou Neel, Ensanya A.He, YanlingPerez, Victor W.Abughosh, Susan M.Hecke, Linde VanPérez-Martínez, P. J.Ackermann-Liebrich, UrsulaHeederik, DickPérez-Zepeda, Mario UlisesAdachi, KristinaHeffron, Raphael J.Perkins, Tracy E.Adams, JamesHege, AdamPerry, Simona L.Adamson, GeorgeHeidmann, IrisPerryman, Lara AnneAden, BrandonHeikinheimo, O.Perry-Parrish, CarisaAdetona, OlorunfemiHeim, JohnPersell, DeborahAdhikari, AchyutHeim, NoorPersinger, MichaelAditjandra, Paulus TeguhHeinemeyer, GerhardPersson-Waye, KerstinAdler, IlanHeld, AndreasPérusse, MichelAgarwal, PriyankaHellewell, SarahPesavento, FrancescoAggazzotti, GabriellaHendrieckx, ChristelPetach, HelenAgostino, GnassoHendrikx, PascalPeter, RichardAgusa, TetsuroHendryx, MichaelPeters, PatrickAhamed, YasminHenes, Sarah T.Peters, SusanAhiablame, LaurentHenkler, FrankPetersen, AnneAhlgren, Erik O.Hennessy, DwightPetersen, KristinaAhmad, Hafiz AnwarHennig, Benjamin D.Petkova, ElisavetaAhmed, Mohamed M.Hennig, FraukePetridis, PanagiotisAkamani, KofiHenrickson, MarkPetrone, PatrizioAkbari, Mohammad RezaHenstra, DanielPetrovský, EduardAkhtar, YasminHeo, Dae SeogPetrusca, DanielaAkk, GustavHeredia-Ortiz, RobertoPezzuto, AldoAkushevich, Igor V.Herian, MitchelPfau, Jean C.Alam, MeerHerman, KatyaPfeffer, KarenAlbrecht, HuguetteHermesz, EditPfinder, ManuelaAlbrecht, JulieHernandez Caceres, Jose LuisPfoertner, Timo-KoljaAlbrecht, Urs-VitoHernandez, AuraPhillips, CarlAlcantara, EnnerHerrenkohl, ToddPhillips, CeriAlegria, Henry A.Herzog, HaroldPhillips, James F.Alemany, SilviaHeuberger, Roschelle A.Phongsavan, PhilayrathAlessandrini, Ester RitaHeusinkveld, HarmPicado-Santos, LuisAlexander, DavidHeuss-Aßbichler, SorayaPichini, SimonaAlexandri, GeorgiaHicks, AlexanderPicó, YolandaAlfa, MichelleHidalgo-Mazzei, DiegoPierce, JessicaAlfonso, MoyaHiguchi, MitsuruPiernas, CarmenAlirezaie, MarjanHil, Allan GPierse, NevilAllais, FlorentHill, ErinPihet, SandrineAllareddy, VeerajalandharHill, HollyPileggi, SilvanaAllebeck, PeterHilton, Thomas F.Pillai, Vijayan K.Allegaert, KarelHimmelgreen, David A.Pillay, YoganAllen, Norrina BaiHind, KarenPinchevsky, GillianAllender, StevenHines, LisaPinto, JosephAlmeida, Maria JuditeHininger-Favier, IsabellePiotrowski, CarolineAlonso-Magdalena, PalomaHinyard, Leslie J.Pira, EnricoAlteren, JohanneHiraishi, AkiraPirard, CatherineAlves De Oliveira, Beatriz FátimaHiroshi, JonalPires, José Carlos MagalhãesAlves, CláudioHirvensalo, MirjaPiringer, MartinAlves, ElisabeteHiscock, RosemaryPirog, Edyta CatalinaAmarasena, NajithHissler, ChristophePirogova, ElenaAmasheh, SalahHitlan, Robert T.Pisaniello, DinoAmenta, Pietro RobertoHjelm, KatarinaPischke, ClaudiaAmini, RezaHjorth, PederPiscopo, VincenzoAmireault, SteveHladish, ThomasPistolesi, MarcoAmiri, AzitaHlaing, WayWayPitkanen, TarjaAmirpour Haredasht, SaraHmielowski, JayPitman, AndyAmram, OferHo, Sai YinPlain, Karren M.An, Kai-NanHock, EmmaPlass, DietrichAn, KyungehHoepner, Lori APlavcová, EvaAnastasiou, DimitrisHoerr, RobertPoetsch, AnsgarAnastasiou, EleftheriosHoff, RodneyPokorn, MarkoAndersen, Christian P.Hoffman, KatePolanska, KingaAndersen, Helle R.Hoffmann, KristinaPolasek, OzrenAndersen, JohnHofman, JellePolimeni, AntonellaAndersen, OttoHofman-Caris, RobertaPollack, AnnaAnderson, Barbara A.Hogan, AnthonyPollmann-Schult, MatthiasAnderson, Stacey EHogan, KarenPolo, InmaculadaAnderson, Todd A.Holick, Michael FPolosa, RiccardoAndino, Jean M.Holloway, Marjan G.Poluzzi, ElisabettaAndrasik, Michele PeakeHolloway-Beth, AlfredaPolya, DavidAndreoli, RobertaHolme, JornPonce Terashima, RafaelAndreotti, GabriellaHolt, Nicholas L.Ponzetto, AntonioAnestis, MichaelHolvik, KristinPopat, RitaAneziris, OlgaHomma, TetsuyaPopoola, LekanAngadi, SiddharthaHon, CarolPorsius, J.T.Angelici, Maria CristinaHonda, AkikoPortengen, LützenAngelo, HillaryHondula, DavidPostels, DouglasAngermann, JeffHong, YouweiPotischman, NancyAnjum, ReshmaHood, Darryl B.Pottie, KevinAnsmann, AlbertHoppe, Liesa KatharinaPoulter, DamianAntar, AlliHoque, Mohammad A.Pourret, OlivierAntoine, BertilleHoque, ShamiaPourshahidi, KirstyAntolini, FrancescoHorney, JenniferPovey, AndrewAntonanzas, FernandoHorta, AnaPowell, CharlesAntoniou, ConstantinosHotta, YoshihiroPowell, LamonteApolzan, JohnHou, DeyiPowell, TaraAppadoo, Srimantoorao S.Houck, Philip DPower, Thomas G.Apparicio, PhilippeHoudmont, JonathanPratt, SaradApsley, AndrewHough, RupertPrensner, JohnArab, AliHouston, Mark C.Prigent, AmélieAraujo, RosaHoward, David C.Prince, BennieArce, LourdesHoward, JustinePringault, OlivierArcos González, PedroHoward, NatashaProchaska, John D.Ard, KerryHowell, Andrew J.Procter, Sandra B.Ardito, LorenzoHowell, Brittany RProdinger, Peter M.Ardjmand, EhsanHribar, LawrenceProskocil, Becky J.Aristieta, AsierHrubec, TerryPruett, Marsha KlineArjunan, ArunHsia, JasonPrzekop, PeterArmstrong, LauraHsieh, Ming-KunPsaltopoulos, DimitrisArola, AnttiHsieh, Nan-HungPuglisi, EdoardoArola-Arnal, AnnaHsieh, Ping-HungPulkrabova, JanaAronson, BenjaminHsieh, Yen-PingPulster, ErinArora, SushrutHsieh, Yi-ChenPunch, Jerry L.Arrebola Moreno, Juan PedroHsu, Hao-JenPurmehdi, MostafaArtiola, Janick F.Hsu, Hui-ChuanPurves, RichardAshford, Nicholas A.Hsu, Lewis Li-YenPuska, PekkaAshour, Mohamed-BassemHsu, Shu-ChienPuska, PekkaAsimakopoulos, Alexandros GeorgeHsueh, Yu-MeiPuy, JaumeAssari, ShervinHu, Ching-YaoPuzzolo, ElisaAsselin, Barbara L.Hu, GuoqingQi, YanAsztemborska, MonikaHu, XuefengQia, FengxiangAttfield, MichaelHua, XinQiao, XiliangAuerbach, PaulHua, YumeiQin, BoAuerbach, SanfordHuang, AntonQin, QuandeAustin, Claire C.Huang, CunruiQin, YanwenAvent, MinyonHuang, Dan LianQiu, JianwenAvogo, Winfred AweyireHuang, GordonQiu, RuofengAydede, Sema K.Huang, Po-ChinQu, XiaoboAydin, AniHuang, QianshengQueirós, AlexandraAzevedo, Juliana AntunesHuang, Xiao (Eric)Quemerais, BernadetteBing, XieHuang, YueQueralt, IgnasiBaack, MichelleHuang, ZhongweiQueralt, IgnasiBabich, MichaelHuber, Stefan AQuick, HarrisonBacker, Charlotte DeHudson, Darrell L.Quirmbach, DianaBackstrand, Jeffrey R.Huelsen, TimRaul, Zamora-RosBacopoulou, FloraHuen, KarenRabito, FeliciaBacosa, Hernando PactaoHughes-Stamm, ShereeRaboni, MassimoBae, Jong-MyonHuitsing, G.E. (Gijs)Rachele, JeromeBaethge, ChristopherHung, Dong-ZongRadecki, JerzyBagheri, NasserHung, Mei-ChuanRadziemska, MajaBagley, Erika J.Hung, Yung-TseRafaj, PeterBaglieri, AndreaHursthouse, AndrewRafalson, LisaBai, WeiHurtado, AnaRage, EstelleBai, YangHussein, TareqRaghupathi, WullianallurBailo, Bibiana GarciaHutchinson, JayneRahman, AtaurBaïz, NourHutton, CraigRajaee, MozhgonBaker, JulienHwang, JunghoRajakaruna, NishantaBakhireva, Ludmila N.Hyder, AyazRajnarayanan, RajendramBakulski, Kelly M.Hynie, MichaelaRaj-Reichert, GaleBalanay, Jo Anne G.Iacovides, HarrisRajtor, MonikaBaldasano, Jose MaríaIan, PaganoRaković, DejanBalderacchi, MatteoIdicula, Titto TRakshit, SudiptaBaldi, ElisabettaIdler, Ellen L.Ralf, RisserBaležentis, TomasIeropoulos, Ioannis A.Ram, YaelBaliatsas, ChristosIglesias-Linares, AlejandroRamey, Andrew M.Balica, Stefania FlorinaIgnacio, JeanetteRamezani, MohsenBaliraine, FrederickIhara, Emily S.Ramirez, OscarBallert, CarolinaIhle, AndreasRamírez-Santana, MurielBalocco, CarlaImeson, AntonRamis, RebecaBalwicki, ŁukaszImpellizzeri, Franco M.Ramos, Carlos AntonioBanay, RachelIndig, DevonRamos-Monserrat, MaríaBanerjee, AreenIngensand, JensRamsahoye, BernardBanerjee, TaraswiInnocent, GilesRamsay, JulianaBanik, Gouri RaniInomata, SatoshiRamström, LarsBani-Sadr, FirouzéInoue, RyoRamyaa, RamyaaBansal, ShwetaInoue, TakeshiRanadheera, C. SenakaBantel, CarstenIonescu, AnisoaraRandall-Simpson, JanisBantjes, JasonIonescu, DannyRandler, ChristophBaragetti, AndreaIossifidou, EleniRanmuthugala, GeethaBaram, ShaharIrene, van KampRantakokko, MerjaBarbe, Anna GretaIrizarry, JavierRantakokko, PanuBarcelos, AnabelaIrvine, IanRao, FengBarcelos, GustavoIsaac, ShabtaiRaskin, LeonBarnard, YvonneIsaacson, MichalRasmussen, Jon B.Barnes, Brendon R.Ishak, SherifRato, Luís Pedro FerreiraBarnes, PhilipIshikawa, NaokoRaven, MelissaBarnett, AdrianIshimi, YoshikoRay, AndrewBarnett, AnthonyIslam, SyedRaymond, ÉmilieBarnett, RossIsler, Robert B.Raynal, AnneBarnish, MaxwellIsokpehi, RaphaelRazul, AzadBarreira, João C. M.Istivan, Taghrid SReady, ElizabethBarrett, Emily S.Ito, TsukasaReady, Paul D.Barrios, CristobalIvan, John N.Reagan, LouiseBarros, NillaIvell, RichardReale, MarcellaBarsanti, SaraIvers, RebeccaReames, Tony G.Barter, Philip J.Ivers, RebeccaRebelo, PauloBarton, ChristopherIzzo, VivianaReddy, Rajyashree N.Barton, Hugh A.Jaacks, LindsayReddy, SandeepBartram, JamieJackisch, ChristianReed, Edward JustyBasset, David R.Jackler, RobertReed, NickBasu, PratyushaJackson, Keith E.Reefhuis, JennitaBathrellos, George D.Jackson-Morris, AngelaRehkopf, DavidBatista, Bruno LemosJacobs, David E.Rehm, Colin D.Battista, RebeccaJacobsen, Jens Kristian SteenReifsnider, ElizabethBaumann, LindaJacobson, SandraRemião, FernandoBaxter, LisaJacquemin, BenedicteRemias, StephenBayat, SaharJahangiri, ArashRen, JingZhengBayer, CharleneJahns, LisaRen, ZefangBeamer, PalomaJaine, RichardRene, Eldon R.Beardsmore, CarolineJalava, KatriRenna, PaoloBeck, Bolette DanielsJames, Katherine A.Resnik, David B.Beck, Kathryn L.James, KathyRestivo, Francesco MariaBecker, JulieJames, SametReszka, EdytaBeckham, JeanJan, Tong RongRetico, AlessandraBeckman, LindaJanssen, IanReynolds, AmyBeer, KarlynJanssen, sabineRhea, SarahBegonia, Maria F. T.Jansson, JohanRibeiro Kvalbein, LuisaBeil, KurtJansson, MäritRibeiro, HelenaBeizaee, ArashJao, JenniferRibeiro, IsabelBelay, BrookJardine, CindyRibeiro, Rita Cláudia CardosoBell, AndrewJarnstrom, HelenaRibisl, KurtBell, LynneJarvandi, SoghraRicceri, FulvioBell, Sarah L.Jarvis, MartinRiccio, PaoloBell, SimonJay, Jennifer A.Rice, LaShanta J.Bell, StephenJayawardene, Wasantha P.Rice, Penelope A.Bellucci, RobertoJeffries, Marlo K. SellinRice, Robert H.Bełtowski, JerzyJendritzky, GerdRichards, Kyle A.Beltran-Bech, SophieJenkins, Jill A.Richardson, GemmaBelviso, ClaudiaJennings, VinieceRicketts, MitchBelyaev, IgorJensen, Per AnkerRiddell, MichaelaBenard, VickiJensen, Randy L.Rider, CynthiaBend, JackJeon, SangchoonRietbergen, Martijn G.Benden, MarkJeong, Jae-WookRietra, ReneBendixen, MonsJeong, JeeyonRigaud, Anne SophieBen-Dov, Iddo Z.Jeremić, SanjaRiggs, ElishaBenedetti, MauraJernigan, David H.Rikard, R.V.Beneduci, AmerigoJesdale, BillRinaudo, Jean-DanielBenjamin-Garner, RubyJho, Hyun JungRioux, Christine L.Benmarhnia, TarikJia, Yunyan AndreaRitchie, JennyBennett, JulieJiang, BinRitchie, KimberlyBenowitz, NealJiang, ChengshengRitchie, StephenBenter, ThorstenJiang, NanRivas, LuciaBerchialla, PaolaJiang, NingboRiveira, Inés SantéBerecki-Gisolf, JannekeJimenez, FelipeRoberts, Jennifer D.Berezin, EitanJiménez-Ballesta, R.Roberts, MeganBerg-Beckhoff, GabrieleJin, AndrewRoberts, RebeccaBerger, KathrynJin, DiRoberts, Sheila J.Bergeron, KimJin, FanRoberts, Stephen M.Berkemeyer, ShomaJin, YanRobichaud, AlainBerkhof, JohannesJin, YongliangRobins, AlanBerkowsky, RonaldJoakim, ErinRobinson, Leslie A.Berlin, Kenneth D.Joe, J. RichelleRobinson, LisaBerman, TamarJoergensen, TorbenRobinson, Scott E.Bernert, RebeccaJohansson Jørgensen, MarianneRobles, LukeBernstein, Eve R.Johansson, BirgittaRobson, Mark GregoryBerry, MeredithJohn, Deborah H.Roca, MartaBerry, PeterJohn, Rita MarieRodas, Juan DavidBertone, EdoardoJohnson, Glenn S.Roderick, PaulBessa, LucindaJohnson, WilliamRodrigues, MartaBesser, JohnJohnston, JillRodríguez, FernandoBethea, JaneJollant, FabriceRodriguez, JoseBettini, SimonaJonathan, SteffensRodríguez-Couto, SusanaBeutell, Nicholas J.Jones, AndrewRodriguez-Garcia, GonzaloBeyer, KirstenJones, AndyRoehling, StefanBhalla, NikhilJones, EmilyRohayem, JuliaBhaskar, Maruthi SridharJones, LaurenRoig-Navarro, Antoni FrancescBianchi, FabrizioJones, Miranda R.Roizen, Jeffrey D.Bianchi, MarinaJones, Rodney PRoma, ElisaBiavasco, FrancescaJoo, Nam-seokRomagna, GiorgioBidasee, KeshoreJordan, JenniferRomanelli, Maria NovellaBielajew, CatherineJorens, Philippe G.Romano, Megan EBierens, JoostJörg, FrederikeRomer, DanBikram, SubediJose Manuel, Rodriguez-LlanesRomero Martínez, ÁngelBilgili, ÖzgeJoy, EdwardRomero-Muñoz, DanielBilotta, PatríciaJukes, DavidRonan, Kevin R.Binfet, John TylerJulian, TimRönnegård, LarsBinyamin, JacquelineJumas-Bilak, EstelleRoque, CarlosBirgand, GabrielJuonala, MarkusRoque, FátimaBiron, CarolineJurewicz, JoannaRosa, Angela Daniela LaBisanz, JordanJuszczak, LesławRosa, Maria JoséBixler, EdwardJutla, Antarpreet S.Rosário, RafaelaBiziuk, MarekJyh-fei, LiaoRosborg, IngegerdBlackall, PatrickKabbani, Toufik A.Rose, ChadBlackmore, CarinaKabisch, NadjaRose, JedBláha, LadislavKaczynski, AndrewRosen, Mitchel A.Blanca, Esther Garcia-TuñonKadam, UmeshRosengren, BjörnBlanco, JuanKaddoura, IhabRoset, JaimeBlaschke, PaulKafoury, Ramzi M.Rossbach, BerndBlasiak, RobertKagawa, TakahideRossi, René M.Bleck, BertramKahlor, Lee AnnRossini, Paolo M.Blesius, LeonhardKaiser, Lucia L.Rothman, DaleBlixen, CarolKakulas, FoteiniRou, Andres DeBlood-Siegfried, JaneKällén, BengtRounds, StewartBlum, Jason L.Kamardeen, ImriyasRousham, EmilyBlumer-Schuettea, Sara E.Kamidis, NikolaosRouten, Ash C.Boardman, JedKamigaki, TaroRowa-Dewar, NenehBoccio, Dana EKaminski, Karol AdamRowlinson, SteveBochner, RosanyKaminsky, TatianaRoy, PrathikBodeau-Livinec, FlorenceKamsu-Foguem, BernardRoy-Engel, AstridBodell, LindsayKanarek, MartyRoy-Gagnon, Marie HélèneBodin, TheoKanarek, Norma F.Ruan, XiangboBoeckmann, MelanieKane, Douglas D.Ruden, DouglasBogdanovica, IlzeKane, EleanorRuiz, FacundoBogen, Kenneth T.Kang, DongmugRuiz-Casares, MónicaBohac, Dave L.Kang, JaekuRuiz-Gómez, Miguel J.Bohme, Diethard K.Kang, JosephineRuiz-Narváez, Edward A.Boldemann, CeciliaKang, Kyung PyoRumchev, KrassiBollati, ValentinaKang, SanghoonRuscin, MarkBolton Moore, CarolynKang, SeungmoRuscio, DanieleBomberg, MalinKano, MegumiRush, BonnieBonari, EnricoKaplowitz, Stan A.Rusmin, RuhaidaBonaventure, AudreyKappell, AnthonyRussell, CharlesBond, BertKapusta, JozefRussell, KellyBond, Vincent C.Karabeg, DinoRussell, TanyaBone, AngieKarabetsos, EfthymiosRusz, JanBonetta, SilviaKarakitsios, SpyrosRutkowski, MelanieBoniface, SadieKaranika-Murray, MariaRutten, EricaBonin, TimothyKaranis, PanagiotisRyan, Kristen R.Bonzini, MatteoKarlic, HeidrunRyan, MichaelBooker, JordanKarlsen, RandiSaad, MohamadBoomer, Jonathan S.Kasedde, SusanSaajanaho, MillaBoon, Helen J.Kasneci, EnkelejdaSaari, Rebecca K.Boone-Heinonen, JanneKatayama, HajimeSabariego, CarlaBooth, AlisonKatner, Adrienne L.Sachmechi, IssacBooth, HelenKatsaounou, ParaskeviSacristan, DanielBooth, JosieKatsouyanni, KleaSadanaga, TsuneakiBorelli, ViolettaKatuchova, JanaSaddik, BasemaBorena, WegeneKatz, Ira.Sadrizadeh, SasanBorja, Judith BKatz, JoanneSaez, MarcBörjesson, StefanKatzourakis, DiomidisSagebiel, John C.Borrmann, ThomasKauko, LeiviskäSagiv, SharonBortsov, AndreyKaunas, Roland R.Sagnelli, EvangelistaBos, ElskeKaushal, NavinSaha, SanjibBosman, CarylKaut, OliverSahay, BikashBothe, DeniseKawa, ArkadiuszSahoo, Prasan KumarBouamra, OmarKawamoto, RyuichiSaint-Maurice, Pedro F.Boucher, OlivierKazakopoulos, PavlosSaito, KanBoudrez, HedwigKe, JingSakai, ToshioBoulware, David R.Keane, EimearSakakibara, MasayukiBourgeois, Denis M.Keegel, TessaSakan, Sanja M.Bovet, PascalKeen, PatriciaSakata, MasahiroBowman, LeighKeevil, StephenSakhaee, KhashayarBownik, AdamKeller, Anita C.Sakhi, Amrit K.Boyas, Javier F.Keller, MarkusSakkiah, Suguna DeviBoyce, James K.Keller, SanSaksena, SummetBoyer, TreavorKelly, David L.Sakurai, EiichiBrachfeld, Stefanie A.Kelly, LeslySala, MariaBrackbill, Robert M.Kemperman, AstridSaleem, MuhammadBracke, PietKennedy, BrianSalemi, Jason L.Bradbury, RichardKennedy, RyanSalgado, PauloBradley, JessicaKennedy, Ryan DavidSalmerón, DiegoBraeye, ToonKennel, JulieSalmond, JenniferBrandt, Diane E.Kerkhove, Maria D. VanSaloniemi, AnttiBråtveit, MagneKeung, EmilySalter, Erica K.Bravaccio, CarmelaKew, OlenSamdal, OddrunBraverman, Eric R.Khalil, ChristianSampson, JohnBreckenridge, Jenna PKhalil, NailaSanchez, Nancy P.Breitner, SusanneKhan, IzharSanders, Alison P.Bricard, DamienKhan, NaserSandle, TimBrinthaupt, Thomas M.Khan, UzmaSandstrom, GillianBrocklehurst, ClarissaKhanal, RakeshSansom, GarettBrody, AaronKhanna, SavitaSantamaria, RitaBronstein, JeffKharma, Mohamed YasserSantilio, AngelaBronzaft, ArlineKhoury, ColinSantos Zanchetta, MargarethBrookes, VictoriaKhoury, PeterSantos, Ana Paula Pires DosBrookfield, KatherineKhuder, Sadik A.Santos, M. Barreiros DosBroom, LainKhush, RanjivSantos, Maria JacintaBrough, LouiseKiesslich, TobiasSantos, Rafael M.Brown, AndrewKilian, ReinholdSantos-Gallego, CarlosBrown, Clayton H.Kim, BumseokSantosh, JatranaBrown, JulieKim, Dae JungSantulli, CarloBrown, KatherineKim, DukhyeonSanz, Maria JoseBrown, PhilKim, HyeongsuSaoud, KhaledBrown, Richard J. P.Kim, Hyung JinSargent-Cox, KerryBrowne, MarilynKim, InhiSarpong, Daniel F.Browning, SteveKim, InkyeomSarrà, MontserratBruschi, Viola MariaKim, Jin-BomSarver , EmilyBrussoni, MarianaKim, John H.Sass, JenniferBrymer, EricKim, Jung-HaSatake, MasayukiBryne, PatrickKim, KarenSathiakumar, N.Buchanan, RobertKim, Sun-YoungSaurina, CarmeBuchanan, SusanKim, Tae IlSauvaget, CatherineBuchanich, Jeanine M.King, Jessica L.Savickaite, RutaBuechler, ChristaKini, UshaSavino, GiovanniBueno Cavanillas, AuroraKinnberg, Karin LundSavolainen, PeterBunch, Martin J.Kinnunen, Tarja InkeriSaxena, NavratiBundschuh, MircoKippler, MariaSaxena, PreetaBünger, JürgenKirchengast, SylviaSaxena, VikasBungum, TimothyKircher, ManfredSaxinger, GertrudeBunt, Craig R.Kirilenko, AndreiSayón-Orea, CarmenBuonfrate, DoraKirkeleit, JorunnSbaffi, LauraBurgason, KyleKirken, Robert A.Sbihi, HindBurgess, John A.Kirsch, ThorstenScales, MonicaBurkart, KatrinKirshen, Paul H.Scarfì, MariaBurke, Rita V.Kirtley, OliviaSchaefer, JeffraBurke, Ronald J.Kirves, KaisaSchaefer, KlausBurrus, RoxanneKitajima, MasaakiSchaffner, Donald W.Bus, James S.Kittner, Jens M.Schechter, Julia CorwinBusardo, FrancescoKjerulff, KristenSchechter, MichaelBusby, ChrisKlaperski, SandraScheepers, PaulBush, KathleenKlarin, BengtScheers, HansBussolati, OvidioKlaschka, UrsulaScheffran, JürgenButterworth, MelindaKlauer, Sheila G.Scheibe, SusanneButtiglieri, GianluigiKlein, Jonathan D.Scheier, Lawrence M.Buunk-Werkhoven, Yvonne A. B.Kleiven, SveinSchembari, AnnaBuxton, JaneKlenke, ReinhardSchijven, JackBuyuksonmez, FatihKlimkowicz-Pawlas, AgnieszkaSchijven, Jack F.Byrne, MiriamKlöckner, ChristianSchiller, GeorgByrne, RebeccaKnisel, ElkeSchipani, StefaniaCaballero, PabloKnott, RachelSchiza, Sophia E.Cabello, MariaKnox, Claire ConnollySchlaff, Rebecca AnnCabral, João P. S.Knudsen, Thomas B.Schlesinger, SabrinaCabral-Pinto, Marina M. S.Ko, Byung-JoonSchlesinger, WilliamCadzow, Renée B.Ko, Ming-ShengSchlumpf, MargretCaesens, GaetaneKobayashi, NobumichiSchmalisch, GerdCalaf, Gloria M.Kobori, YoshitomoSchmalwieser, Alois W.Calavalle, Anna-RitaKoch, FlorianSchneider, AlexandraCalderón-Garcidueñas, LilianKoch, ManjaSchnell, IzhakCalderón-Larrañaga, AmaiaKociu, ArbenSchock, HelenaCallison, KevinKodzius, RimantasScholkmann, FelixCalvo, Ana I.Koenig, Mary DawnSchöpfer, JuttaCalvo, ConcepciónKoenraadt, Sander CJMSchreinemachers, DinaCameron, JillKöeper, BirgitSchultz, StephenCampbell, Daniel B.Kohlbacher, FlorianSchulz, Peter J.Campbell-Arvai, VictoriaKöhle, Maria PapathomaSchumacher, SarahCampos, Carlos J. A.Kohno, KunieSchutte, AnneCan, ArnaudKok, EstherSchwebel, DavidCanales, RobertKok, GerjoSchwitzguebel, Jean-PaulCanha, NunoKolovelonis, AthanasiosSchwitzguébel, Jean-PaulCantonwine, David E.Kondo, MichelleSciomer, SusannaCao, ShiKono, RyoheiScott, PippaCao, YangKonstantinov, Spiro M.Scott, Theresa L.Cao, YongXiaoKontopantelis, EvanScrafford, Carolyn G.Caplan, LeeKopp, MartinScrucca, FlavioCardoso, LuísKorhonen, IlkkaScullin, Michael K.Cargo, MargaretKormas, KonstantinosSearle, Amelia K.Carignan, Courtney C.Korpela, Kalevi M.Sebilo, MathieuCariñanos, PalomaKorzeniecka-Kozerska, AgataSeckmeyer, GuntherCarlin, DanielleKorzeniewska, EwaSee, Lai-ChuCarling, Philip C.Kósa, KarolinaSeedorf, HenningCarlisle, Gretchen K.Koshy, KoshySegura-Ortí, EvaCarll, AlexKosmider, LeonSeitz, ChristopherCarloni, ElisaKoszycki, DianaSekhar, SonalCarlos Alberola, MarKotrschal, KurtSekine, YoshikaCarlsen, Hanne KrageKotsakis, Georgios A.Sell, KatieCarlsson, AxelKoupenova-Zamor, MilkaSellix, MichaelCarlsson, GunillaKousha, TermehSelya, ArielleCarmichael, Amtul RazzaqKouvonen, AnneSembajwe, GraceCaron, FrançoisKovacic, Melinda ButschSemmens, ErinCarpenter, DavidKoyama, AsukaSenwo, ZacharyCarré, VincentKoyama, YuSereni, FabioCarrera, JenniferKozlowski, Michael R.Serralheiro, Maria Luísa M.Carreras, HebeKraft, Anke Renate MariaSerrano, FernandoCarrero, Jose AntonioKraft, MichaelServidio, RoccoCarrier, MathieuKrall, Jenna R.Servien, RémiCarroll, ClintKramer, AxelSetti, IlariaCarter, Ellison M.Kramer, LouisaSetyan, AriCarter, HollyKrata, Agnieszka AnnaSha, ZheCarter, KimberlyKratzke, CindyShah, NilayCartier, ClémentKrieg, EdwardShah, TayyabCarugno, MicheleKrieger, BobShahdah, UsamaCaruso, BethanyKrienert, Jessie L.Shaheed, Mohammad SaadCaruson, KikiKristiansen, JesperShalaby, Ahlam I.Casanovas, CarmenKristjansson, Alfgeir L.Shalaby, Mohamed SamehCasas, IreneKroesen, MaartenShama, Ayman. A.Caserta, DonatellaKroll, AlexandraShamasunder, BhavnaCasey, JoanKruize, HannekeShang, CeCasilari Pérez, EduardoKrukowski, HenrykShao, ChaofengCasotti, RaffaellaKruse, GinaShao, KanCassels, SusanKrushkal Adkins, JuliaShapiro, DeborahCastañeda, CarmenKrysinska, KarolinaShareck, MartineCastellano, LeandroKuipers, MirteSharma, JagannathCastillo-Michel, HiramKuipers, PimSharp, LindaCastrillo, PaolaKukreja, SubhashSharpe, Richard M.Castro, MarisolKulaga, ZbigniewSharples, JasonCather, CorinneKumar, SanthoshShavelle, Robert MCattaneo, AndreaKumpel, EmilyShaw, JamesCavalline, TaraKundi, MichaelShaw, LisaCazenave, AnnyKunst, AntonShea, KylaCeaser, DonovonKuo, KirstyShea, Patrick J.Ceola, SerenaKuo, Mu-HsingShearer, MartinCervero Aragó, SílviaKuo, Shu-lungSheehan, Mary C.Chai, JianyuanKuo, Yu-MingSheinkopf, StephenChai, RifaiKurir, Tina TičinovićShen, JiabinChakraborty, MuktaKurppa, SirpaShen, WenzhongChalkias, ChristosKuwahara, YasutakaShen, WinnyChamberlain, JimKvaal, KariShen, XueyouChambers, EarleKvítek, LiborShepherd, DanielChambers, StephanieKwak, Moon K.Shepherd, JohnChamine, IrinaKwan, Christine ManlaiSherafat-Kazemzadeh, RoyaChan, Benny Kwok KanKwan, Mei-PoSherchan, SamendraChan, Chee HonKwok, AlisonSherratt, FredChan, Daniel K. Y.Kydonaki, ClaireSherrington, CatherineChan, GregLa Rocca, Renato V.Sheu, Dwan-FangChan, Heng Choon (Oliver)Laabir, MohamedShi, ChunmingChan, Janet Kit YanLabouliere, Christa D.Shi, LizhengChan, Ka ChungLaCaille, LaraShi, LuChan, Karen Kar LoenLadeira, CarinaShi, QiangChandran, ArunaLado, FrédéricShi, TiezhuChanel, OlivierLadomerský, JurajShi, YunfengChang, Chia-JungLaestadius, LinneaShields, LindaChang, Chih-ChingLafrenie, RobertShiff, CliveChang, Chih-HungLahham, AdnanShigemura, KatsumiChang, Hsin-ChiehLahortiga, FranciscaShimizu, AtsushiChang, Hsin-YuanLai, Hung-YuShin, Hyeon-ShicChang, Hsueh-shengLai, Ka ManShin, Seung GuChang, Hui-huaLai, PeggyShin, Won SikChang, KaowenLai, Shih-WeiShirakawa, HiroakiChang, Kee-lungLaidlaw, MarkShirure, VenkteshChang, Seo IlLaing, IngridShive, SteveChang, Ta-YuanLaird-Offringa, Ite A.Shiyomi, MasaeChang, Yi-tangLaliotis, IoannisShneyderman, YuliyaChang, ZhengLAM, Nicky Y. F.Shoji, KensukeChangyoon, JeongLam, Tsan Yuk TommyShoji, KotaroChapman-Novakofski, KarenLaman, Jeffrey A.Shortle, WalterCharles, KatrinaLambertini, ElisabettaShowell, ChrisChastang, Jean-FrançoisLamina, ClaudiaShrestha, VinishChatterji, SomnathLamontagne, TonyShu, AnpingChatzidiakou, LiaLan, Shu-Jan J.Shvetsov, Yurii B.Chau, K. W.Lane, Charles R.Siahpush, MohammadChaudhry, Qasim AliLane, KevinSiceloff, E. RebekahChe, HuizhengLang, ChristinSiddiqi, KamranChe, YueLang, JessicaSiddiqui, Muhammad A.Chelbi, SoniaLanger, SarkerSidiropoulos, PantelisChen, BingLangiano, ElisaSiebeneck, LauraChen, BoLangley, TessaSiebenmann, ChristophChen, ChaoqiLangrish, Jeremy P.Sieber, NancyChen, Cheng-NanLanier, YzetteSiegel, PamelaChen, Chi-FengLantz, AndrewSiegel, RobertChen, FengLanzola, GiordanoSifunda, SibusisoChen, GuoQianLao, Xiang-qianSigmund, ErikChen, JiangangLapanje, AlešSignorelli, SalvatoreChen, JiaxuLarsen, Ann DyreborgSilla, Lucia Mariano Da RochaChen, JinLarsen, Douglas P.Silva, LígiaChen, Jui-ChiLarsen, MarkSilveira Pinto, RafaelaChen, Jui-ShengLau, Ho Yin EricSimeonov, Vasil D.Chen, Jung-WeiLaunes, CristianSimini, FilippoChen, Kow-TongLaursen, BjarneSimmering, Jacob E.Chen, Mei-RuLauterbach, JohnSimona, CandianiChen, Pei-ShihLava, Sebastiano A. G.Simonds, VanessaChen, QiangLavado, RamonSinclair, MarthaChen, Ruei-mingLavagna, MonicaSinclair, RyanChen, Ruey-YuLavalliere, MartinSinger, Andrew C.Chen, Solomon Chih-ChengLavallière, MartinSingh, HarsimranChen, WeihongLavezzi, AnnaSingh, KamaleshwarChen, Wei-HsiangLavia, LisaSingh, Nirbhay N.Chen, WeiweiLavie, Carl J.Singh, TusharChen, Wendy Y.Lavoie, Raphael A.Singhrao, Sim K.Chen, YaoningLaw, Anandi V.Sint, Tin TinChen, Yi-ChunLawler, JennySironi, SelenaChen, Yi-MingLawn, SharonSirtori, CarlaChen, YurenŁawniczek-Wałczyk, AnnaSiskind, VictorCheng, Eddie W. L.Lawrence, ClaireSissung, Tristan M.Cheng, June J.Laws, Michael BartonSit, ReginaCheng, Kai-WenLawson, Joshua A.Sjödin, FrederikCheng, Shyh-YuehLawson, JustinSkaaby, TeaCheong, Hae-kwanLe Dé, LoïcSki, ChantalChernyak, SergeiLe Hénaff, MatthieuSkjølsvold, Tomas MoeChernyavskiy, PavelLe, Thi Thu YenSkwarzec, BogdanChéron, EmmanuelLeal Pacheco, Fernando AntónioSlack, TimCherukupalli, RajeevLeary, EmilySleeth, DarrahCheung, Darren Man-waiLebelo, Sogolo L.Slingsby, AidanChiang, Chien-HsiehLee, Chang SooSliwa, SarahChiang, Hung-LungLee, Chen-ChenSloan, Robert AlanChiang, Shu-henLee, ChristinaSluiter, JudithChiang, Tai-anLee, Ho-WonSluiter, JudithChien, Chun-RuLee, HueiSly, TimothyChien, Ling-ChuLee, Huei-JaneSmethurst, PhilipChien, Lung-ChangLee, James J.Smiley, SabrinaChien, Yeh-ChungLee, JosephSmith, Elizabeth R.Chih, Ming-YuanLee, Min-GeolSmith, GaryChileshe, NicholasLee, MiryoungSmith, MelodyChilibeck, PhilLee, Nora L.Smith, ThomasChillón, PalmaLee, Paul H.Smith, Woutrina A.Chin, MichaelLee, Pei-ChenSodoudi, SaharChipps, JenniferLee, Pyoung-JikSoellner, RenateChitchumroonchokchai, JulieLee, Seok-LyongSolbiati, MonicaChiu, Cheng-HsunLee, Sue Ann S.Solcan, CarmenChiu, Melvin W.Lee, Wai-LingSole, MontserratChliaoutakis, JoannesLee, Wallis A.Solís-Guzmán, JaimeCho, Hyun-HeeLee, Wing-keeSolomon, ColinCho, Yoon HeeLee, Yau-JiunnSolomon, PattyChoi, Byoung WhuiLEE, Yiu Yin RaymondSoloski, Mark J.Choi, Doo-SupLee, Yung-JaanSolovieva, YuliaChoi, Hyun-DeokLee, ZinaSolovyev, NikolayChoi, Jae WookLees, Rosemary SusanSone, MichihikoChoi, KelvinLefering, RolfSoneja, SutyajeetChoi, Won S.Leffondré, KarenSong, GuoChong, Ka ChunLegros, AlexandreSong, JinxiChou, Chin-ChengLehner, AngelikaSoo Chung, DooChou, Yu-ChingLei, MeiSoudeyns, HugoChristinet, VanessaLeidner, AndrewSousa, ArturoChristou, PaulLeire, CharlotteSouza, SofiaChrysikopoulos, Constantinos V.Leitão, AnaSouza-Junior, Tacito P.Chrzanowski, ŁukaszLeitzmann, MichaelSovacool, BenjaminChu, Ching-PingLemke, Lawrence DSparks, RossChu, Chun-HsiaoLenoble, VéroniqueSpeed, ShaunChu, Chun-HungLeón, GerardoSpence, NicholasChua, Tze-ErnLeonardi, GiovanniSperlich, BirgitChuang, Cheryl CYLeondiadis, LeondiosSperry, BenChuang, Hsiao-ChiLeone, AntonioSpeybroeck, Alexander VanChuang, Hung-yiLeoni, EricaSpickett, JefferyChuang, Kai-JenLepeule, JohannaSpiezia, LucaChuang, Li-MinLeslie, Frances M.Spilak, Michal ProctorChuang, Ting-WuLessner, LawrenceSpilsbury, James C.Chummun, HarryLetcher, ToddSpinazzè, AndreaChun, BumseokLetts, CarolynSpinney, JamieChun, Kwok PanLeung, JanniSponder, MichaelChung, Hsin-FangLevallois, PatrickSpratt, DanielChung, Il-MoonLeventhall, GeoffSprunger, JoelChung, JackyLevitan, RobertSrivenugopal, KalkunteChung, Min-HueyLevitan, Robert D.St. Hilaire, Melissa A.Ciarkowska, KrystynaLevy, Andrew P.Stabile, LucaCiccacci, CinziaLevy, StevenStack, StevenCiccone, Marco MatteoLewis, BradleyStafoggia, MassimoCihacek, LarryLewis, RyanStahl Jr., Ralph G.Cisneros, RicardoLewison, GrantStalsberg, HelgeCizmas, LeslieLi Wai Suen, ConnieStam, RianneClaborn, DavidLi, BaiStamatakis, MichailClaggett, BrianLi, BaoguangStamatiadis, NikiforosClar, ChristophLi, ChaoyangStamatis, NikolaosClark, Maggie LynnLi, ChuanrongStanifer, John W.Clark, Terryann CoralieLi, FadongStanistreet, DebbieClark, Thomas A.Li, HaochuStarling, Anne P.Clarys, PeterLi, JinhuiStarr, Martha A.Claßen, ThomasLi, Kevin W.Staskiewicz, GrzegorzClatts, Michael C.Li, KuanyuStavrinos, DespinaClawson, AshleyLi, LanjuanStearns, DianeClay, KarenLi, LeahSteels, StephanieCliff, DavidLi, LimingSteffy, DavidClifford, AngelaLi, MiaoStefler, DenesClifton, PeterLi, QianjinStein, AlfredClough, AlanLi, QingfengSteiner, Jennifer L.Cochran, BlakeLi, Rita Yi ManSteinle, SusanneCoelho, J. Luis BentoLi, ShandySteinmaus, CraigmCoenen, MichaelaLi, SiweiStella, SimoneCoffield, EdwardLi, TiantianStellman, Jeanne MagerCoffman, MakenaLi, WeidongStepanov, IrinaCohen, Chari A.Li, WeijuanSterling, DavidCohen, Steven A.Li, XiaodongStevens, Gregory D.Cole, DonaldLi, XiujunStewart, Anna MColeman, Blair N.Li, YangStewart, JillColeman, TimLi, YingSticherling, MichaelCollie, RebeccaLi, YipingStichnothe, HeinzCollings, PaulLian, Ie-BinStiehl, EmilyCollings, PaulLiang, BinqingStifter, JanetCollins, Mary B.Liang, Ching-PingStingone, JeanetteColombo, ClaudioLiang, YiruiStockings, EmilyConceição, Eusébio Z. E.Liang, ZhenglunStöger, ReinhardConcettina, FengaLiao, Chia-WenStorti, Kristi L.Conlon, Kathryn C.Liao, Chien-senStowell, AlisonConnolly, DeirdreLiao, QiŠtrac, Dubravka ŠvobConrad, AmyLiao, Wen-LingStrandh, MattiasCornejo-Ovalle, MarcoLiao, YungStrazdins, LyndallCornelis, Marilyn C.Liapi, CharisStreckert, JoachimCortés, UlisesLichtveld, MaureenStretesky, Paul BCortese, ClaudioLie, ArveStrickland, Matthew J.Cortese, FrancescaLieberman, Lauren JoyStriley, Catherine W.Corvini, PhilippeLilenfeld, LisaStrong, CarolCosma, AlinaLiller, KarenStroshine, MeghanCosta, PaulLilley, RebbeccaStruck, SylviaCostanzo, SimonaLilley, RebbeccaStübiger, GeraldCoughenour, CourtneyLim, DavidStumpf, AstridCoull, Bruce M.Lim, HyeyeunSu, JosephCoupland, Lindsay. J.Lin, Ann C.Su, MeirongCousins, RosannaLin, BrendaSu, ShiliangCoussens, ScottLin, Chang-ShenSu, Ta-ChenCoutts, ChrisLin, Chih-ChuanSubbiah, MuruganCoxson, PamelaLin, Gen-MinSugimura, HaruhikoCoyne, IainLin, Huann-shyangSui, XuemeiCraig, JeffLin, Jen-JiaSullivan, TrudyCramb, SusannaLin, Ken-YuSumner, WaltonCrawford, Sybil L.Lin, Ming-YengSun, DandanCrews, John E.Lin, Pao-HwaSun, DanfengCriado-Alvarez, Juan JoseLin, YanSun, HansenCrippa, PaolaLin, Yi-ChinSun, HaoCrisafi, FrancescaLin, Ying-HsuanSun, JianCrisman, Thomas L.Lin, Yi-PinSun, LongshengCritchley, JuliaLin, Yuan-ChungSun, MingCrocombe, LeonardLin, Zong-HongSun, QinghuaCronin, AidanLincoln, JenniferSun, SunCrouse, Dan L.Lindberg, Ann-SofieSun, WenjieCrouse, Dan LawsonLinder, StephenSun, ZhaonanCrowe, Brandi M.Lindquist, MarkSundell, JanCui, JianLindsay, RyanSundermann, ErinCukier, SamanthaLinks, JonathanSuner, SelimCullinan, PaulLiorni, IlariaSuñer-Soler, RosaCummings, K. MichaelLiou, Jiann-hawSung, JidongCunha, AngelaLipner, Shari R.Sunindijo, Riza YosiaCunningham, KendaLipshultz, Steven E.Suppan, PeterCunningham, Solveig ArgeseanuLithgow, BrianSuppes, LauraCuriel-Esparza, JorgeLitonjua, AugustoSureda, XiscaCurl, Cynthia L.Little, JulianSurma, MagdalenaCurtis, JackieLiu, Chen-YuSussan, ThomasCurtis, Jacqueline W.Liu, Chia-ChyiSutherland, Matthew T.Cushing, LaraLiu, Chien-TsaiSutton-Grier, ArianaCycoń, MariuszLiu, HongliangSuwazono, YasushiCyrys, JosefLiu, JianSuzuki, KohtaD’Agostino, StefaniaLiu, JianSuzuki, Yuichiro J.Da Cunha, Kenya Moore DiasLiu, JinglingSvartengren, MagnusDa Silvab, Joaquim C. G. EstevesLiu, Kevin Fong-ReySvob, ConnieDąbrowska, JolantaLiu, RuiSwanson, JohnDahl, ÅslögLiu, SiminSwartz, MelissaDai, Chia-YenLiu, TingSylvetsky Meni, AllisonDai, JiapeiLiu, VivianSzabo, AmandaDailianis, StefanosLiu, Wen-ChengSzáková, JiřinaDakhama, AzzeddineLiu, XiaoyuSze, TonyDalvie, Mohamed AqielLiu, XuefengSzulczewska, BarbaraDamashek, AmyLivingston, MichaelSzumska, AgnieszkaDamiani, GianfrancoLleò, Maria M.Szumski, GrzegorzD’Amico, FabrizioLlirós, MarcSzyszkowicz, MieczyslawDaniel, BillLo, ChristopherSzyszkowicz, MieczysławDaniell, WilliamLo, Huang-MuTa, Van M.D'Antona, GiuseppeŁobocki, LechTadashi, ToyamaDanz, NicholasLoboda, AgnieszkaTaebi, BehnamDanziger, Larry HLoddenkemper, RobertTaghizadeh, NiloofarDarby, KateLodovici, MauraTaillie, Lindsey SmithDarnton, AndrewLoh, MirandaTain, You-LinDarques, RégisLohner, SzimonettaTakahashi, KenDashti, HassanLohwacharin, JenyukTakahisa, KanekiyoDasmahapatra, AsokLoke, Alice YuenTakamura, NoboruDautovitch, NatalieLollar, DonTalavera-Garcia, RubenDavey, RachelLönnberg, StefanTalbot, PrueDavis, MaryLoonen, RoelTalhout, ReinskjeDavlasheridze, MeriLopez Jornet, PiaTaliaferro, LindsayDawkins, LynneLopez, Maria J.Talib, Latif MohdDe Andrade, DeniseLopez-Fernandez, OlatzTally, StevenDe Blas, MaiteLorenzo, Trinidad OoteroTam, Cheuk-ChiDe Boer, LuitzenLoughran, SarahTamminen, Manu V.De Bor, Margot VanLouis, Valérie RTamura, AtsutakaDe Brito, JorgeLourenço, J.Tan, LiDe Camargo, Olaf KrausLoury, SharonTan, MinghongDe Castro Nery, Susana Vaz Da SilvaLovallo, William R.Tanaka, HiromitsuDe Castro, Shamyr SulyvanLovell, RebeccaTanaka, JunkoDe Donato, FrancescaLovreglio, PieroTanaka, NobuhikoDe Gruijl, FrankLowenberger, CarlTandy, SusanDe Guise, ÉlaineŁozowicka, BożenaTang, JianjunDe Jong, SimoneLu, ChuanwenTang, NaijunDe Kinderen, ReinaLu, GuangquanTang, TiantianDe Lourdes Pereira, MariaLu, JianTang, YingzhongDe Mestral Vargas, CarlosLu, Tsung-hsuehTanihara, ShinichiDe Miguel, EduardoLu, WenhuaTanner, GritDe Palma, GiuseppeLu, XiwuTannous, KathyDe Paula, Fabiana MartinsLu, ZhongbingTansey, Malú G.De Pinho, Joana RoqueLuben, TomTao, HongDe Re, ValliLuby, Stephen P.Tao, YanDe Santis, RobertoLucas, RichardTappin, DavidDe Sario, ManuelaLucchese, GuglielmoTappin, DavidDe Vries McClintock, Heather F.Lucht, Jan MTar, JozsefDean, Lorraine T.Lucifora, ClaudioTarantino, GiovanniDeGuzman, Pamela B.Lugo, AlessandraTarima, SergeyDeHart, DanaLui, Edmund M. K.Tarman, KustiariyahDehner, Corey DenenbergLuís, Ana TeresaTartakovsky, L.DeJarnett, NatashaLuk, John M.Taskinen, SeppoDekker, LindaLukasiewicz, AnnaTaube, JoeDeLellis, NailyaLull, Melinda E.Tausk, FranciscoDelgado, JordiLund, SandyTaylor, CarolineDelgado, MelvinLundqvist, AndreasTaylor, GemmaDelgado-Calle, JesusLuo, JinTaylor, GemmaDelisle, HélèneLuo, MingTaylor, Kira C.Dellinger, AnnLuo, TaoTaylor, LizDelmelle, Eric M.Lusa, MerjaTaylor, TimDelmonico, MatthewLushington, KurtTe Riele, HeinDelpla, IanisLusk, AnneTebbens, Radboud J. DuintjerDelucchi, Kevin L.Luz, Paula MendesTedla, Yacob G.Demeritt, DavidLyall, KristenTefe, MosesDemirev, PlamenLynagh, MaritaTegner, YelvertonDemyanets, SvitlanaLynch, Robert A.Teixeira, SamanthaDeng, YanpingLyon, MaureenTejedor-Alonso, Miguel A.D'Errico, Maria NicolàLyons, BeverlyTeli, DespoinaDesapriya, EdiriweeraM. Greene, CatherineTempesta, MariaDeSesso, John M.Ma, GuanshengTemple, Norman J.Deth, Richard C.Ma, HaitaoTenenbaum, David ElliotDetzel, PatrickMa, LuTeng, JingDeumlich, DetlefMa, XingmaoTennekes, Henk A.Deurwaerdère, PhilippeMa, YanboTenta, RoxaneDevine, SueMacArthur, GeorginaTeodoro, Cordova-FragaDevy, JérômeMacauley, MatthewTerčelj, MarjetaDeWitt, Jamie C.MacCarthy, MichaelTerry, DanielDi Ciaula, AgostinoMacCarthy, SarahTervonen, HannaDi Folco, Maj-BrittMacleod, CatrionaTerzaghi, ElisaDi Giovanni, GeorgeMacMillan, FreyaTesche, MatthiasDi Lazzaro, VincenzoMadhuku, MorganTestoni, InesDi Luca, MarcoMadimenos, Felicia C.Teti, DianaDi Martino, FerdinandoMadoff, Lawrence C.Thanavala, YasminDi Palma, LucaMadrigano, JaimeThangamani, SaravananDiaz, AlejandroMaekawa, FumihikoThe, Natalie S.Diaz, JulioMagiera, TadeuszTheofilatos, AthanasiosDickerson, Aisha S.Magnavita, NicolaTheorell, TöresDiep Yeung, Cassandra S.Magnussen, Costan G.Theorell, TöresDifranza, JosephMaguire, AnneTherrell, BradfordDihl, Rafael RodriguesMagzamen, SherylThielens, ArnoDijk, J. P. (Jitse) VanMaher, ChristopherThiery, MickaelDijkstra, GerardMaher, Jaclyn P.Thoma, MyriamDildy, Gary A.Maher, WilliamThomas, EvanDillon, LindseyMaheu-Giroux, MathieuThomas, HollyDing, Gang-QiangMahmud, HasanThomas, Lisa C.Ding, TianbingMahoney, MaggieThomas, Roger E.Dinkel, DanaeMailey, EmilyThomas, Stephanie MargareteDirinck, EvelineMainka, AnnaThomas, StuartDirks, Kim NatashaMaisonet, MildredThomas, Timothy K.Dismukes, AndrewMaitland, CloverThomée, SaraDissanayake, CherylMaître, AnneThompson, DeborahDixit, VinayakMajeed, BanThorne, KymDixon, DanielMaksymiuk, GabrielaThornley, GenevieveDjukovic, DanijelMalaguarnera, MicheleThorpe, ClareDodson, ZanMalalgoda, ChamindiThunders, MichelleDoerr, CelesteMaldonando, Julie K.Thurston, GeorgeDohle, SimoneMalecki, KristenThurston, George D.Doiron, AmberMaleku, AratiTian, LinweiDolejská, MonikaMalemud, Charles J.Tian, YilinDollman, JamesMalhotra, PraveenTiller, DanielDolnicar, SaraMaluk, CristianTing, Cheh-ShyhDomínguez-Vilches, E.Mamen, AsgeirTinggi, UjangDonath, LarsMamudu, Hadii M.Tippett, VivienneDong, ChunjiaoMancl, Karen M.Tiranti, DavideDonini, AndrewMandic, SandraTobollik, MyriamDonne, BernardMangwandi, ChiranganoTochkov, KirilDonovan, CynthiaMannetje, Andrea’tToderi, StefanoDoos, LucyManning, LouiseToft, GunnarDoran, NealMannucci, Pier MannuccioTokonami, ShinjiDorea, CaetanoManor, IrisTolley, Elizabeth A.Dórea, José G.Manso, José R. PiresToloo, SamDorosh, AndriyMansouri, KamelTomata, YasutakeDörr De Quadros, PatriciaManzo, CiroTombs, SteveDotzauer, AndreasMao, AimeiTomita, HaruyoshiDougherty, Michael JohnMao, LiangTommasi, ToniaDrace, KevinMao, LiminTommasino, LuigiDragon-Durey, Marie-agnesMarć, MałgorzataTon, Shan-ShinDrakos, AndreasMarchand, AlainTonetti, LorenzoDrancourt, MichelMarchant, BenjaminTong, Hoang V.Draper, PeterMarchbank, KevinTong, RuipengDreassi, EmanuelaMarcilla, AntonioTong, ZhemingDries, JanMarcus, MichaelTong, Zhong HuaDriver, KaraMAREMMANI, ICROTonin, StefaniaDrolet, Jean-PhilippeMargui, EvaTonn, BruceDrummond, Mark A.Marin, D.E.Toppila, EskoDrummond, NeilMarino Reis, Amélia PaulaTordeux, AntoineDrydakis, NickMariottini, Gian LuigiTorres Lagares, DanielDu Laing, GijsMarkey, SeanToscano, William A.Du Prel, Jean-BaptistMarkov, Marko S.Tota-Maharaj, KiranDuan, WenjieMarkowski, VincentTovar, AndresDuckham, BryanMarks, EricTownsend, JulieDuclot, FlorianMaršálek, BlahoslavTownsend, Sarah S. M.Duddu, Venkata RamanaMarselle, MelissaTrabace, LuigiaDugas, DanielMarsh, LeighTran, Huy N.QDukie, ShailendraMarshall, NadineTran, Ngoc HanDulipovici, Alina MariaMarston, LouiseTran, PamelaDumas, OrianneMartín, CarlosTranchant, CaroleDumbaugh, EricMartin, RyanTravelet, ChristopheDunning, TrishaMartin, UnaTraversa, AmarantaDuntas, LeonidasMartinelli, LucaTrawley, StevenDurand-Zaleski, IsabelleMartinez Dominguez, FranciscoTrehan, IndiDurgo, KsenijaMartinez, AndresTremblay, MarkDuriez, ElodieMartinez, VictorTronchin, LambertoDurkin, AnneMartinez–Arguelles, Daniel B.Trucco, ElisaDurkin, HelenMartinez-Gimeno, AntonioTruehart, AmberDusso, AdrianaMartínez-Graña, AntonioTrulli, EttoreDuzinski, Sarah V.Martins, MariaTruong, KhoaDyer, James S.Maruf, Abdullah AlTsagarakis, KonstantinosDziubanek, GrzegorzMarzano, LisaTsai, I-ChunEadson, WillMarzban, LucyTsapakis, IoannisEakin, Michelle N.Mascart, GeorgesTseng, C.-M.Eastin, Matthew D.Maschke, ChristianTseng, Kuo-HsinEbi, Kristie L.Masieri, FedericaTseng, Ming-LangEbisu, KeitaMason, Lisa ReyesTso, For YueEddyani, MiriamMason, RobertTsourtos, GeorgeEdgerton, JasonMasood, MohdTsuji, JoyceEdokpayi, JoshuaMassarsky, AndreyTu, Wen-lingEdouard, PascalMassey, Rachel I.Tuão Gava, Carlos AlbertoEdwards, Joshua R.Masterman-Smith, HelenTucker, JohnEdwards, LoreceMasters, NicoleTurenne, Christine Y.Edwards, MichaelMatanoski, GenevieveTurner, LyleEdwards, PeterMathers, Amy J.Turner, Scott G.Effertz, TobiasMatheson, KimberlyTurner-Cobb, JulieEfird, JimmyMathews, ShinyTurrell, GavinEfird, JimmyMathieu, LucTuszynski, JackEgawa, ShinichiMathur, HarshTutino, MariaEgli, ThomasMathuthu, MannyTwigg, LizEguchi, HisashiMatsubara, KeiichiTwyman, LauraEisenbarth, HedwigMatsukura, KeiichiroTymvios, NicholasEkman, BjörnMatsumura, KentaTyson, JulianEkmekcioglu, CemMattingly, Stephen P.Tzamalouka, GeorgiaEkundayo, OluMattson, Mats-OlofTzeng, Huey-MingEl Bcheraoui, CharbelMattsson, SörenTzoraki, OuraniaEl Ghaziri, MazenMatus, Patricia Isabel CorreaUchikoshi, YuukoEl Khalloufi, FatimaMatysiak-Klose, DorotheaUddin, ShahadatEl Shahawy, OmarMatzarakis, AndreasUgedo, LuisaElgeneidy, AhmedMatzke, Gary R.Ulatowski, LynnElhag, Dhia EldinMaupomé, GerardoUlibarri, GerardElliott, Michael B.Mavridou, AthenaUnger, Joseph M.Ellis, Kelsey N.Mavrot, FabienUr Rehman, MasoodElton-Marshall, TaraMawson, AnthonyUusi-Rasi, KirstiEmanuel, Amber S.May, FelicityUzan, PierreEmili, Lisa A.Mayer, Brooke K.Uzu, GaëlleEmond, Claude.Mayner, LidiaVaarama, MarjaEnemark, UlrikaMays, Darren MichaelVacchina, VéroniqueEngelmann, GuidoMazumdar, SoumyaVácha, RadimEngesser, Karl-HeinrichMazumdar, SoumyaVadenbo, Carl O.Enocsson, HelenaMazumdar, SumitVähäkangas, KirsiEnomoto, MasaruMazumdea, DiyaVakulin, AndrewEpton, TracyMazur, JoannaValentukevicien, MarinaErazo, MarciaMazzacane, SanteValič, BlažErdur, HebunMbengue, SaliouVallero, DanielErmler, SibylleMbulo, LazarousVallini, GiovanniEronen, JohannaMcbean, GordonVallmuur, KirstenEsmaeili, BehzadMcCann, ClareVallone, Donna M.Espinosa, Hugo G.McCarthy, HelenValmaggia, Lucia R.Esposito, CiroMcCarthy, Mark J.Valverde, PatriciaEsposito, SergioMcCauley, DarrenVan Ballegooijen, WouterEštoková, AdriánaMcclain, Craig J.Van Boven, Job FMEttekal, IdeanMcClure, JohnVan Bruggen, ArienaEttema, DickMcCormack, LaceyVan Cauwenberg, JelleEvans, HalaMccowan, LesleyVan Cauwenberg, JelleEvans, Josie M.M.McCoy, Sarah J. BreeseVan Den Berg, JuliaEvans, MarilynMcCreesh, NickyVan Den Bosch, MatildaEvans, RichardMcDermott-Levy, RuthVan Den Ende, JefEvans, Sarah FMcDonald, JamesVan Der Heijden, JeroenExadaktylos, AristomenisMcDonald, Yolanda JaneVan Der Hoek, WimEykyn, Thomas R.McDonnell, AndrewVan Der Meeren, AnneEysel-Gosepath, KatrinMcEgan, RachelVan Der Steen, HansEzaki, TakahiroMcElrath, KarenVan Der Wel, Kjetil A.Ezendam, JanineMcGrail, Matthew M.Van Diepeningen, A.F Kresina, ThomasMcGuire, SarahVan Dijk, Meine PieterFabbri, KristianMcIsaac, Kathryn E.van Halem, DorisFabbricino, MassimilianoMckay, RuthVan Heerde, Waander L.Fabris, LindaMcKenzie, EricaVan Hoof, JoostFactor-Litvak, PamMcKenzie, LisaVan Os, Bertil J. H.Fairchild, GeoffreyMcLachlan, MichaelVan Rienen, UrsulaFairlie, IanMclain, JeanVan Rossem, LenieFakhr, MohamedMcLennan, Ian S.Vance, EricFalkinham III, JosephMcLeod, GeraldineVance, Marina E.Fallacara, Dawn M.McLeod, Robert D.Vaportzis, Eleftheria (Ria)Falone, StefanoMcleroy, KenVárbíró, GáborFalster, KathleenMcLuskey, JohnVardoulakis, Sotirisfan, hongqiangMcMurran, MaryVargas, JorgeFan, QiMcNally, RichardVárhelyi, AndrásFang, Guor-ChengMcNamara, MaireadVarotto, Silvia F.Fang, JianguoMcPhaul, Kathleen M.Vásquez, ElizabethFang, KaiMcQuistan, Michelle R.Vaughn, MichaelFang, QinhuaMead, Erin L.Vaz Moreira, IvoneFang, XiuqiMearns, KathrynVaz-Moreira, IvoneFang, YaMedek, DanielleVedøy, Tord FinneFanizzi, Francesco P.Medina, SylviaVeeranki, PhaniFantke, PeterMehmood, AmberVeiga-Lopez, AlmudenaFärber, HaraldMehra, AradhanaVeliziotis, MichailFarber, Harold J.Meier, RobertVelotti, LuciaFarhat, GhadaMeints, KerstinVelten, MarkusFarhat, GraceMeisel, FrankVenetsanou, FotiniFarmer, John G.Melander-Wikman, AnitaVentura, CarloFarnleitner, AndreasMelkani, GirishVentura, NatasciaFarrar, MarkMelquiades, Fábio LuizVenturini, ElisaFarsalinos, KostantinosMeng, LinghuiVera, LuisaFasano, EvelinaMericle, AmyVerd, SergioFassbinder, JoergMerkiel, SylwiaVerginelli, IasonFaustini, AnnunziataMertens, JasminVerhaegh, GeraldFavas, PauloMetcalf, PatriciaVerhaeghe, Pieter-PaulFaverjon, CélineMethner, UlrichVerhoeven, Adrie J.Favia, GuidoMetzinger, LaurentVerhoeven, AukjeFawcett, LeeMeyer, StephenVerhoughstraete, MarcFawkner, Samantha G.Meyerholz, DavidVerlicchi, PaolaFedele, FrancescoMeynet, PaolaVerloigne, MaïtéFeingold, Beth J.Mezei, GaborVerropoulou, GeorgiaFeng, LeiMeziti, AlexandraVesper, StephenFeng, XinglinMiao, AijunVetter, Stefan W.Feng, ZhanchunMichanowicz, AndrewVexler, AlbertFeng, ZhongxiangMichelozzi, PaolaViazzi, FrancescaFenlason, Lindy ChristineMidura, Ronald J.Viegas, CarlaFenton, ThomasMiech, RichardViegas, SusanaFernandez-Lopez, HelenaMiglio, RossellaVierheilig, JuliaFernández-Pachón, María SoledadMignon, Sylvia I.Vigeh, MohsenFernández-Robledo, José AntonioMihailova, SandraViguié, CatherineFerrante, MargheritaMikler, Armin R.Vijayalaxmi, VijayalaxmiFerreira, AdelinoMill, Jose G.Vijayan, MuraliFerreira, Carla Sofia SantosMiller, CharlesVila, MercedesFerreira, LuisMiller, Daniel N.Villano, MichaelFerreira, SaraMiller, J. MitchellViolanti, John M.Ferrero, EnricoMiller, MarkVirani, ShamaFerris, JasonMiller, NickVita, RobertoFerronato, ChiaraMiller, Rachel L.Vitali, MatteoFervers, BéatriceMiller, ShellyVliet-Ostaptchouk, Jana VanFicco, GiorgioMiller, William P.Voaklander, Donald C.Fieber, LynneMillwood, IonaVogel, UllaFielding, JaneMina, I Aguiar-PintoVolberg, Rachel A.Figueiral, Maria HelenaMinardi, DanieleVolker, BeateFigwer, JarosławMínguez-Alarcón, LidiaVolkova, LiubovFindlay, DavidMinisola, SalvatoreVolland, GerhardFindlay, RobertMiraglia, Simone Georges El KhouriVollmer, SebastianFindorff, Mary J.Mirca, ZottiVollrath, MarkFine, James BurkeMirlohi, SusanVon Lewinski, DirkFinegood, DianeMirman, Jessica HVon Lindern, EikeFiner, SarahMirzaei, ParhamVoon, PaulineFink, AstridMishra, JayshreeVose, SarahFink, GabrielMishra, ManishVosvick, Mark A.Finka, AndrijaMitra, RaktimVuorio, AlpoFinkenauer, CatrinMitsiakos, GeorgeWachter, Jan K.Finlay, Warren H.Miyagawa, MuneyukiWackowski, OliviaFinley, BrandonMizukawa, HazukiWadzinski, ThomasFinn, SymmaMlynarek, StevenWagatsuma, YukikoFiocchi, SerenaMnatzaganian, GeorgeWagenbrenner, NatalieFiocco, AlexandraMo, LingfeiWagner, JeffFirmino, HoracioMöckel, ThomasWagner, MartinFischer, GabrieleModesti, Pietro A.Wagstrom, KristinaFischhoff, BaruchMoffatt, Cameron R. M.Wahls, TerryFisher-Hoch, Susan P.Mohajeri, NahidWainwrigh, MiltonFisk, MalcolmMohammad, SaidWal, Jillon S. VanderFitzgerald, EdwardMohammad, Saif M.Walcek, ChrisFitzgerald, NeilMolina, Ana IsabelWałdykowski, PiotrFitzsimmons, Eric J.Molina-Navarro, EugenioWalia, HarneetFiúza, AntónioMoliner-Urdiales, DiegoWaliszewski, Stefan M.Fizka, MichaelMoll, Henri C.Walker, Ana Paula PimentelFlacke, JohannesMøller, Jens KjølsethWalker, ThomasFlamand, ClaudeMoncada, StefanoWallace, DavidFlander, LouisaMondal, DebapriyaWallace, MaeveFleet, JamesMondal, Nandan KumarWallace, Susan E.Flemming, Hans-CurtMonge, María EugeniaWaller, KimFleury, Marie-JoséeMonninkhof, EvelynWallington, Timothy J.Flex, AndreaMontgomery, Marilyn ChristinaWallis, Belinda A.Florek, EwaMontuori, PaoloWalls, AngusFlorios, KostasMonyeki, KotsediWallston, Kenneth A.Floros, GeorgiosMoore, NathanWalorczyk, StanisławFluegge, KyleMoore, Simon C.Walsh, Anne MarieFogarty, AndrewMorabito, MarcoWalsh, JohnFOK, LincolnMoraga Serrano, Paula EstherWalters, AnnikaFoos, BrendaMoraga-Serrano, PaulaWalters, DianneFoote, Janet A.Morales Suarez-Varela, MariaWalters, MatthewFord, AllisonMorales, JulioWalton, AnnMarieFord, JudyMoreda-Piñeiro, JorgeWalz, RogerFord, Michael T.Morell, StephenWalz, YvonneForsythe, K. WayneMoreno, Ricardo D.Wan, NengFortier, Michelle A.Moretto, AngeloWang, BeiFoss, Y.J.Morey, Lisa M.Wang, Chi-ChuanFoster, Kenneth R.Morgado, Mariza G.Wang, Chong-WenFoster, WarrenMorgan, Ian G.Wang, DaowenFourtounas, CostasMorgan, JimWang, FahuiFoust, Jr., Richard D.Morin, CoryWang, GangduoFox, ERIN R.Morisset, Anne-SophieWang, GuanghuaFraile, RobertoMoriya, ShingoWang, HaitianFrancesco, AlettaMorrell, Christopher H.Wang, Han-HsiangFrancis, JoelMorris, BrendanWang, HaoFranco, Noelia GarciaMorris, BrianWang, HeFranco, RodrigoMorris, MelanieWang, JianliFranco-Watkins, AnaMorrison, ChristopherWang, JianxuFranke, ChristineMorrissey, AnneWang, JianyingFranklin, PeterMorrongiello, Barbara A.Wang, Jong-ShyanFranks, Amy MMorrow, JenniferWang, JunboFrans, Van GeerMoschis, George P.Wang, Ming-DongFransen, ErikMosgöller, WilhelmWang, MoFrazer, KateMoshammer, HannsWang, Peng-HuiFrederick, ArmahMosnier, AnneWang, PingFree, AndrewMoss, Elica M.Wang, QunFreeman, Andrew J.Moss, JenniferWang, SenFreeman, TobyMossey, Peter A.Wang, ShengruiFreene, NicoleMou, JinWang, Tai-ChiFrenzel, AnneMouahid, GabrielWang, WeiFrew, EmmaMoustris, Konstantinos P.Wang, XiangyuFriedrich, RainerMoutari, SalissouWang, XinchangFriel, JamesMovahed, AssadWang, XiuhengFriger, MichaelMovsisyan, NarineWang, XuanFristedt, SofiMpofu, EliasWang, YananFroggatt, TerryMudgal, AbhisekWang, YangFromm, BastianMueller, Anna S.Wang, ZhaoliFronczyk, JoannaMueller, Mike FreyaWang, ZhenboFrontistis, ZachariasMuhammad, NAWAZWang, ZhenchaoFrost, David M.Mui, Kwok-waiWang, ZhenshengFuentes, EnriqueMulligan, AislingWang, ZhoujingFuertes, ElaineMulligan, KateWang, ZunyaoFuhrmann, ChrisMulligan, KathleenWant, Elizabeth J.Fujii, YasuhitoMullins, Robert F.Waples, JamesFujii, YoshiakiMulvaney, Caroline A.Ward, LeighFuks, KaterynaMunehiro, KazunoriWard, LesleyFukuda, MisaoMurakami, KeikoWard, MaryFuller, TrevorMuromachi, YasunoriWard, TimothyFunari, ValerioMurphy, Adam B.Warfield, Marji EricksonFung, Ivan Wing HongMurphy, EndaWashbourne, Carla-LeanneFunk, Richard H. W.Murphy, FionaWashburn, JasonFunk, William EMurphy, HelenWasko, DennisFusaro, MariaMurray, EmilyWaterlot, ChristopheGaardbo Kuhn, KatrinMurtaugh, MaureenWatkins, Adam J.Gabbianelli, RositaMusaad, SalmaWatmough, GaryGadepally, Vijay N.Musher-Eizenman, DaraWatson, Gene E.Gadomski, Anne M.Mustapha, AdetounWayadande, AstriGaffo, Angelo L.Muster, TimWayal, SonaliGail, LotharMutowo, MutsaWears,, Robert LewisGaitskell, KeziaMutti, LucianoWebb, AnnGajski, GoranMyklebust, Lars HenrikWebb, ThomasGalagher, JenniferMyong, Jun-PyoWebber, LauraGalán, CarmenNabaweesi, RosemaryWeber, NicoleGalante, FrancescoNachman, KeeveWebster, BonnieGalesloot, TesselNachman, RebeccaWei, BinnianGall, ElliottNagai, MasatoWei, BinnianGallagher, RobynNaganuma, AkiraWei, JunniGallert, ClaudiaNagata, Jason M.Weinmann, SheilaGallopel-Morvan, KarineNagayoshi, MakoWeinstein, MarcGalusha, Aubrey L.Nagelkerke, NicoWeisel, CliffordGamet-Payrastre, LaurenceNahar, Vinayak K.Weitzel, MatthiasGander, JenniferNai Ping, GaoWelch, DavidGao, ChuansiNaidu, RaviWelgamage Don, AakashGao, JunlingNaidu, SathyWeller, Donald E.Gao, MengNair, SaraswathyWells, EllenGao, YangNaish, SuchithraWells, NancyGao, YuNakajima, KazunoriWells, YvonneGarcia, Ada LNakamura, MasashiWelsh, PaulGarcia, Daniel MartinezNakanishi, ShinsukeWernstedt, KrisGarcía, FernandoNakano, JuntaWest, CarynGarcia, ValerieNam, Eun WooWest, RyanGarcía-Martínez, OlgaNan, JiangxiaWestervelt, Daniel M.García-Pérez, JavierNanovskaya, Tatiana N.Weston, KathrynGardiner, PhilipNante, NicolaWeyer, PeterGarrett, BridgetteNardell, EdwardWhite, BrandiGarrido, Marcial VelascoNardone, NatalieWhite, KimGartner, CoralNarsavage, GeorgiaWhitesell, Peter L.Gasana, JanvierNarushima, MiyaWhitmee, SarahGaskin, Darrell J.Nash, Kevin A.Whittaker, PeterGaspar, KrisztianNathanielsz, PeterWibowo, SantosoGasper, John T.Naumovski, NenadWickliffe, JeffreyGast, Richard K.Nawrocka, AgnieszkaWiderström, MicaelGates, MadisonNayak, PratibhaWidiatmojo, ArifGatterer, HannesNduna, MzikaziWieczorowska-Tobis, KatarzynaGavrieli, AnnaNegishi, TomoeWiegner, MatthiasGay, MelvinNegy, CharlesWieland, DianeGe, Ren-ShanNelson, KaraWielgomas, BartoszGe, XiaodongNelson, Kara L.Wiklund Axelsson, SarianneGea, Oliveri ContiNerud, KimberlyWilcox, Rand R.Gebel, ThomasNesson, ErikWilén, Britt-marieGebremariam, Seyoum Y.Neto, Maria AugustaWilkinson, JeremyGeddes, JeffreyNettles, KendallWilliams, Catrin FGeedipally, Srinivas ReddyNeupane, SubasWilliams, D’AnnGeidne, SusannaNewcombe, RhiannonWilliams, W. LarryGeiger, TobiasNewman, Alan P.Willinger, RémyGeisler, MattNewman, John F.Wills, Thomas AGelberg, Kitty H.Neymotin, FlorenceWilson, Debra RoseGemar, MasonNg, Chris Fook ShengWilson, Fernando A.Genchi, ClaudioNg, JasonWilson, LeighGennuso, Keith P.Ng, NawiWinder, Richard S.Gentry, Terry J.Nguendo Yongsi, H.B.Windsor, L. JackGeorge, Tony P.Nichols, Michelle G.Wing, Victoria C.Georgiev, AleksandarNicholson, LisaWinkler, PetrGera, NidhiNickbakhsh, SemaWinneke, GerhardGharbia, SalemNicolas, RemyWinters, CarenaGhorayeb, Sleiman R.Nicolopoulou-Stamati, P.Woith, WendyGhosh, SomiranjanNie, Linda H.Wójkowska-Mach, JadwigaGiaccone, ValerioNie, PengWojtkowska, MałgorzataGianicolo, Emilio Antonio LucaNielsen, KariWolff, MaryGiannaros, Theodore M.Nielsen, Samara JoyWolfsdorf, Joseph I.Giannoni, MartinaNieminen, PenttiWolkoff, PederGibb, HermanNiemitz, Jeffrey W.Won, Young-JooGibbons, AlyssaNiidome, TakuroWong, Danny Ka-HoGibbons, JosephNikolakaki, EleniWong, Grace Lai–HungGibbs, BenNing, ZhiWong, Ling-timGibson, Kristen E.Niu, JingpinWong, Man-SauGibson, LauraNjinga, Ray L.Woo, ShiaoGifford, Janelle A.Nkomo, Vuyisile T.Wood, David W.Gifford, SandraNofer, Jerzy-RochWoodfield, LorayneGilkey, DavidNoguchi, MasaWoods, Heather ClelandGill, Simone V.Nolan, Laura B.Woods, James S.Gill, TiffanyNolan, RachaelWoodward, AlistairGillaizeau, FlorenceNonnemaker, JamesWoody, GeorgeGillet, NicolasNoonan, DevonWoolcock, GeoffGilmore, DaveNorman, TrevorWouters, Eveline J. M.Gilroy, RoseNorrman, JennyWright, James C.Gindi, Renee M.Norval, MaryWright, JimGiné Garriga, RicardNoszticzius, ZoltánWrzosek, MałgorzataGiorgi, GabrieleNoumsi, Ives Magloire KengneWu, ChaoGissler, MikaNourazari, SaraWu, Chung-HsuenGiven, BarbaraNovack, TessioWu, GuangxueGkoulalas-Divanis, ArisNowak, Michal S.Wu, Jer-HorngGlauert, Howard P.Nowak, RobertWu, KangshengGlavan, MatjažNowalk, Mary PatriciaWu, Kevin Chien-ChangGlick, Alexander F.Nower, LiaWu, LianfengGlock, SabineNowicki, MichalWu, LiyunGlow, Steven D.Nriagu, JeromeWu, Ming-PingGobbo, Liana C. DelNtougias, SpyridonWu, ShixinGodar, Dianne E.Nucci, Luciana B.Wu, TongningGodfrey, Donald A.Nunes, AnaWu, Tsung-ChihGodfrey, SamNuñez, Maria CristinaWu, Wei-NingGodleski, JohnNunez, NomaliXi, BoGoel, AnujNurius, PaulaXi, JunqiangGoel, RameshNussler, Andreas K.Xia, NingshaoGoggins, William B.Nweke, Onyemaechi C.Xiang, GuoqiangGohlke, JuliaO Cuiv, ParaicXiang, JianjunGoin-Kochel, Robin P.O’kane, MauriceXie, GraceGold, Laura S.O’Sullivan, KimberleyXie, GuoxiangGoldson, EdwardOba, ShinoXie, ShaohuaGoldstein, Adam O.O’Brien, Katie M.Xin, JinyuanGoldstein, SusanO’brien, KellyXing, YalanGolightly, DavidOccelli, FlorentXiong, MayGolka, KlausOcobock, CaraXu, BinGomes, JoãoO'connor, Richard J.Xu, Elvis GenboGómez, XiomarO'Connor, ShawnXu, Feng-LianGomez-Alvarez, VicenteOdland, Jon ØyvindXu, JianzhongGonçalves, GuilhermeOdom, Stephen R.Xu, JinGong, JianhuaOgata, MakikoXu, LinGong, PingOgata, NorioXu, RenGong, ShiweiOgeil, RowanXu, WeilinGonzales, FelisaÖgren, MikaelXu, XiaoguangGonzález, María JoséOh, Chang MoXu, XiaoyueGoodfellow, Lynda T.Oh, NamkyungXu, YanqingGoodin, DouglasO'Hare, AnthonyXu, ZhihengGoodman, PatOhashi, NaroXu, ZhiweiGoosey, EmmaOhwaki, KazuhiroYallapu, Murali M.Goovaerts, PierreOikonen, MerviYamaguchi, NobuyasuGoppelt-Struebe, MargareteOjala, AnnYamamoto, NaomichiGorczynski, PaulOkabe, AtsuyukiYamamura, SoichiroGordon, Catherine A.Okafor, FlorenceYamaoka, ShojiGorgievski-Duijvensteijn, MarjanOkayama, MasanobuYan, BeizhanGorgoulis, Vassilis G.Okoro, Chinyere K.Yan, Wai YeungGorini, FrancescaOkuyama, YasuhideYan, XuedongGorman, ShelleyOkweye, PaulYan, YanchunGorman, ShelleyOlaussen, AlexanderYan, ZhijunGorniak, Stacey L.Oldfield, FrankYang, Albert C.Gorr, MatthewOliveira, José António VasconcelosYang, Cheryl C.H.Górski, JózefOller, AdrianaYang, DujuanGoseberg, NilsOlomu, IsokenYang, FeiGOTO, AzusaOlsen, Robert G.Yang, FengGouda, HazemOlson, David L.Yang, HongGoudas, MariosOlu, OlushayoYang, HongGould, GillianO'Neill, DarraghYang, Kwang IkGovindan, MalarvannanOngerth, JerryYang, LinGraber, JudithOnicescu, GeorgianaYang, MihiGradinaru, Simona RalucaOosterkamp, MargreetYang, QingchunGradoni, LuigiOpdam, NiekYang, TielinGrady, SueOppert, Jean-MichelYang, Tse-ChuanGraham, JayOppliger, AnneYang, XianfengGraham, MelissaOrdonez, AlmudenaYang, XiaomeiGrana, RachelO'Reilly, KathleenYang, XinGranado, M. DoloresOrmandy, DavidYaniv, YaelGrandner, Michael A.Ornelas-Soto, Nancy EdithYao, JieGranieri, AntonellaOrr, NeilYasmin, ShamsunnaharGrant Ludwig, LisaOrr, RobYAU, Yiu HungGrant, WiliamOrtega, JohnYazdani, MazyarGrantz, DavidOrton, SophieYe, FanGrases, FelixOsaki, YoneatsuYeargin, SusanGravel, JocelynOsborne, NicholasYeckel, Catherine WeikartGravely, ShannonOshio, AtsushiYen, Cheng-FangGray, DeborahOskrochi, G RezaYen, Chia-HungGray, DeborahÖst, AnitaYeon-Kyu, KimGray, Heewon LeeOsteen, PhilipYepes, VíctorGray, JenniferOsuna, EduardoYershova, KseniyaGrayson, Dennis R.Ota, AtsuhikoYick, Yee YingGreacen, TimOtsuki, TakemiYiin, Lih-MingGreatbatch, IanOtte, DietmarYi-Kai, JuanGreen, NathanOu, Chun-QuanYin, AitianGreenberg, AlexandraOu, JudyYin, JunGreenberg, MarkOudin, AnnaYing, ZhekangGreenberg, Robert M.Oughton, MatthewYokoyama, YokoGregersen, MereteOuyang, BinYong, Tai-soonGreim, HelmutOuyang, Jian-MingYoo, WonsukGreppi, AnnaOuYang, XiaokunYoshida, TakahikoGriffith, GemmaOviedo-Joekes, EugeniaYoshiharu, FukudaGriffith, LaurenPacheco Torgal, FernandoYoshiki, SyujiGriffiths, PeterPacheco, JuliannaYoshinaga, JunGrimalt, JoanPacheco, MárioYoshinaga, ShinjiGrimsley, L. FayePaci-Green, RebekahYoshioka, EijiGroner, Judith A.Padula, AmyYoshizumi, TerryGronlund, CarinaPadula, Amy MYoul, PhilippaGross, Susan MichellePage, DeclanYoung, HeatherGroten, KarinPaglialonga, AlessiaYounge, SineadGrunig, GabrielePagnini, FrancescoYoussef, Ahmed M.Grzybowska, EwaPal, SandipYu, DapengGrzywacz, Joseph G.Palazzolo, Dominic L.Yu, GangGu, YinaPaleti, RajeshYu, Hoi-Ying ElsieGuarino, HonoriaPalinkas, Lawrence A.Yu, LiangGuarnaccia, ClaudioPalma, CarlaYu, VivianGuauque-Olarte, SandraPalma, GiuseppeYu, Xue QinGuerrero-Preston, RafaelPalmer, Raymond F.Yu, YunxianGuerriero, MassimoPalmer, SherryYuan, QuanGuevara, MarcPalumbo, CarlaYue, YaojieGugusheff, JessicaPan, JiayanZaaijer, Hans L.Guilarte, TomasPan, MeixiaZaborskis, ApolinarasGuirguis, KristenPan, XiaochuanZaidi, M. RazaGulis, GabrielPanagopoulos, Dimitris J.Zalejska-Jonsson, AgnieszkaGulson, BrianPanagopoulos, ThomasZamyslowska-Szmytke, EwaGumbo, Jabulani R.Paneque, PilarZapolski, TamikaGun, RichardPang, XiaobingZarrillo, Marguerite L.Guo, FengPang, YuanjieZavadskas, Edmundas KazimierasGuo, Ling-ZhongPankhurst, MichaelZavar, ELYSEGuran, SerpilPantazaki, Anastasia A.Závodská, AnitaGurley, Emily S.Pantic, IgorZawadzki, JarosławGururajan, RajPao, MARYLANDZeigler-Johnson, Charnita M.Gushulak, Brian D.Papadimitriou, LamprosZelikoff, JudithGustafsson, PeikPapadopoulos, AthanasiosZeng, BiaoGute, David M.Pappas, SteveZeng, ChenGutiérrez, María-joséParajuli, RajendraZeng, DiGyorkos, ChristinaParazzini, MartaZeng, GuangminGyouhyung, KyungPardío-Sedas, Violeta T.Zeng, Xu-HuiHa, HoehunParedes, EstefaníaZeni, NicolaHaan, Mary N.Parise, Carol A.Zhai, GuofangHaas, Gerda-MariaPark, BoyoungZhan, BenjaminHabre, RimaPark, Chan-SikZhang, AiyongHack, JochenPark, HyesookZhang, ChenHains, DavidPark, Jae HyunZhang, ChunxiaHajek, AndréPark, Jeong-HunZhang, ChunyongHakulinen, ChristianPark, JiyoungZhang, DingmeiHale, BeverleyPark, Ki HwanZhang, GuangmingHalford, Nigel G.Park, Sin-AeZhang, HongliangHall, BrittPark, SojungZhang, JieHall, SophieParker, HelenZhang, LibinHalls, JoanneParker, Samantha E.Zhang, LuluHamaguchi, MasahideParkinson, Don-RogerZhang, MinHamano, TsuyoshiParris, Christie L.Zhang, PeihuaHamden, KhaledParrish, Anne-MareeZhang, PengHammal, FadiParrott, WayneZhang, QingHan, Der-ShengParry, CharlesZhang, QingzhuHan, DongmeiParsons, MatthewZhang, RuiliHan, FengxiangPartonen, TimoZhang, ShiqiuHan, JialiPartos, TimeaZhang, TaoHan, MyoungseokParveen, SabihaZhang, TingbinHan, XuemeiPasay, CieloZhang, TingruHan, Young-JiPaschalidou, Anastasia K.Zhang, YifengHance, ThierryPaschall, MallieZhang, YunhuiHang, Yiu KaiPaslakis, GeorgiosZhang, YunlinHanna, Elizabeth G.Passerone, RobertoZhang, YuqiangHannig, JanPassey, Samantha L.Zhang, ZhengdongHänninen, JariPastor-Valero, MariaZhang, ZikeHansen, Anders Blædel GottliebPatelarou, EvridikiZhao, BaoluHanson, AdrianPatrick, RobertZhao, JayHanson, BuckPatterson, FredaZhao, JinzhuoHansson, LenaPatterson, James W.Zhao, XingquanHaque, UbydulPatterson, KristineZhao, YangHara-Kudo, YukikoPatton, Allison P.Zhao, YashuangHarbron, RichardPaul, Christopher JohnZhao, YiHardy, Kristina K.Paul, RichardZhao, YunHargrove, DerekPaulsen, PeterZhao, ZhanHarjumaa, MarjaPaul-Ward, AmyZheng, QiHarlan, SharonPavlinac, PatriciaZheng, TongzhangHarland, JanicePavuk, MarianZheng, YifanHarley, Naomi HalldenPavydė, EglėZhong, WeihongHarri, RintalaPawlowska, BeataZhou, MaiHarrington, JohnPayne, AdaZhou, XiaoluHarris, CristenPayne, Thomas J.Zhou, ZhijunHarris, Curt A.Pearson, MichaelZhu, BaoliHarris, FionaPechlivanis, SonaliZhu, HongHarris, MarkPeck, Richard M.Zhu, RongHarry, KambezidisPeckham, StephenZhuang, Jie (Jackie)Hart, Peter D.Pecorari, ElianaZia, KashifHartley, WilliamPedersen, Arve VorlandZielinski-Gutierrez, EmilyHartmann, James X.Pedersen, EjaZimeri, Anne MarieHarvey, Carol A.Pedisic, ZeljkoZiogas, ArgyriosHassan, SayedPeek, SebastiaanZischg, AndreasHaszard, JillPeel, JenniferZnad, HusseinHatzopoulos, StavrosPei, De-ShengZoeller, ThomasHatzopoulou, MariannePeixe, Tiago SeveroZoppini, AnnamariaHauptmann, MichaelPelclova, JanaZou, WenHauser, PeterPeller, JulieŹróbek-Sokolnik, AnnaHavard, AlysPelletier, GuillaumeZukowski, LisaHawes, StephenPeluso, Marco E. M.Zwoździak, Anna Hawkins, Eric J.Pembrey, Lucy
Hawkins, JaredPeng, Wenbo


